# Deciphering the lithium ion movement in lithium ion batteries: determination of the isotopic abundances of ^6^Li and ^7^Li

**DOI:** 10.1039/c9ra02312g

**Published:** 2019-04-16

**Authors:** Marcel Diehl, Marco Evertz, Martin Winter, Sascha Nowak

**Affiliations:** University of Münster, MEET Battery Research Center, Institute of Physical Chemistry Corrensstraße 46 48149 Münster Germany Sascha.Nowak@uni-muenster.de; Helmholtz-Institute Münster, IEK 12, Forschungszentrum Jülich GmbH Corrensstraße 46 48149 Münster Germany

## Abstract

Lithium ion batteries (LIBs) are the energy storage technology of choice in the context of renewable energies and electro-mobility. It is imperative to get a thorough understanding of the aging mechanisms to achieve a prolonged cycle and calendar life. One major drawback of the technology is continuous capacity fading during operation, which is partly attributed to the loss of active lithium, the object of this work's analysis. While lithium ion battery aging is an intensively researched topic, there is still the need to determine the origin of the lost lithium and the lithium migration into the different cell components over time. To achieve this goal, different plasma-based mass spectrometric techniques in combination with isotope analysis are applied to obtain bulk as well as depth-resolved information about lithium ion movement and distribution of lithium in aged LIB cells. Different aging experiments are performed on NCM622/graphite cells with a ^6^Li-enriched electrolyte with subsequent Li analysis of the cell components. The results show that the charging rate, as well as the cycle number, has an impact on the ^6^Li/^7^Li-abundances and that the overall abundances show a rapid mixing of the isotopic species already after the first charge/discharge cycle for all cell components.

## Abstract 2

Different aging experiments were performed on NMC622/graphite cells with a ^6^Li enriched electrolyte to unravel the lithium distribution.

## Graphical abstract

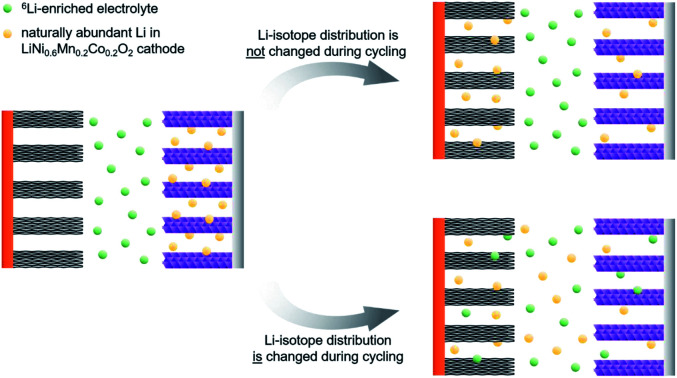

## 1 Introduction

The growing demands for renewable energies and the urge of reducing the human ecological footprint increased the attention towards LIBs for mobile and stationary use.^1,2^ State-of-the-art LIBs use carbonaceous materials (*e.g.* graphites, hard carbon and their composites, such as MesoCarbon MicroBead/hard carbon (MCMB/HC))^3^ and layered lithium transition metal oxides (LiMO_2_; M = Ni, Co, Mn and/or Al) as the negative and positive electrodes, respectively.^4–6^ Commercially used cathode materials are quaternary mixtures – including lithium, nickel, cobalt, and manganese – in various stoichiometries, referred to as NCM.^7^ Additionally, an electrolyte is needed to maintain ionic conductivity between the two electrodes. For this, a mixture of cyclic and linear carbonates – like ethylene carbonate (EC) and ethyl methyl carbonate (EMC) – is used,^8,9^ in which a conductive salt, *e.g.* lithium hexafluorophosphate (LiPF_6_)^10^ is dissolved. However, a major downside of this technology is that it still suffers from a decreasing performance over its lifetime of which the cause is still debated in the literature.^3,11–14^ Consequently, this decline is referred to as “aging” effects of the LIB. One of these aging effects is attributed to the loss of cyclable (“active”) lithium during extended charge/discharge cycling of the LIB either on the negative carbonaceous electrode or positive electrode, resulting in a fading capacity. On the carbonaceous negative electrode, this loss is induced by the highly reductive potentials (0.025 V *vs.* Li/Li^+^) during the charging process leading to the (partial) decomposition of the electrolyte.^15^ This is especially pronounced during the first cycle, whereas a passivation layer is formed, the so-called solid electrolyte interphase (SEI), which prevents the electrolyte from further decomposition.^14,16–20^ While most of the lithium is immobilized (=“lost”) during the first cycle, the SEI can still grow and change during subsequent cycling, which can be induced through other aging effects, *e.g.* transition metal dissolution from the positive electrode.^21,22^

On the cathode side, another interphase forms during cycling, the cathode/electrolyte interphase (CEI).^23–25^ However, the CEI is more than one order of magnitude thinner than the SEI^14,26^ and is much less understood, but it still attributes to the capacity fading and loss of active lithium.{Börner, 2016 #378} In addition to that, there are more reported aging effects that attribute to the fading capacity of the positive electrodes including an increasing impedance, irreversible phase changes of the layered material and the release of oxygen at elevated cut-off potentials.^14,23,27–29^

Although there is a sophisticated repertoire of electrochemical methods for monitoring, the need for additional analytical methods is indispensable.^30–34^ For direct solid analysis, methods like glow discharge-mass spectrometry (GD-MS), laser ablation-inductively coupled plasma-mass spectrometry (LA-ICP-MS), time of flight-secondary ion mass spectrometry (TOF-SIMS) and X-ray photoelectron spectroscopy (XPS).^35–42^ Due to their capabilities of directly measuring the surface components, they represent promising approaches for analyzing new materials as well as the degradation of aged cells. Especially the GD-MS benefits from unique traits such as a fast analysis time while maintaining a nanometric sputter-rate and the ability to perform an isotope analysis throughout the profile of an electrode. Moreover, it is possible to detect a wide range of concentrations simultaneously, as well as a semi-quantitative evaluation without the need for standardized matrices.^43–45^

Nonetheless, the loss of lithium was already reported extensively in the literature, while the origin is still not fully clarified. Here, the use of isotope-labeled materials would help to better understand these losses and tailor future LIB for their needed desires.^14,16,29,46^

In this work, the lithium migration through the cell and the changes during cycling by means of different plasma-based mass spectrometric techniques in combination with isotope analysis are explored by using a ^6^Li-enriched electrolyte. The cell system is based on a NCM622/MCMB(HC) Li ion full cell operated at nominal voltages of 4.2 V using a EMC/EC (1 : 1 by weight) based carbonate mixture as electrolyte solvent. This method in combination with the mass spectrometric analytical techniques opens up manifold possibilities because the two main isotopes of lithium – ^6^Li and ^7^Li – have different abundances depending on the component and changes of these abundances can be measured after cycling. On the one hand, bulk information are obtained for the anode, cathode, and electrolyte using ICP-MS. On the other hand, depth profile analysis is applied to gather sophisticated information about the depth-resolved isotopic abundances of lithium to get a better understanding of the decomposition mechanisms.

## 2 Materials and methods

### 2.1 Electrolyte preparation

For the performed aging experiments an electrolyte system consisting of a 1 M solution of ^6^LiPF_6_ (95 atom% ^6^Li, 98% chemical purity (CP), Sigma Aldrich) in a mixture of ethylene carbonate/ethyl methyl carbonate (EC/EMC, 50/50 (wt/wt), 22 μL L^−1^ (measured) H_2_O, 99.9%, Solvionic) was prepared in an inert argon atmosphere (<0.1 μL L^−1^ H_2_O, <0.1 μL L^−1^ O_2_). After dissolving the salt in the solvent, the mixture was stirred at room temperature using a magnetic stirrer at 450 rpm for 12 h. Additionally, a commercially available electrolyte with the natural isotopic abundance of lithium and the same mixture of carbonates (EC/EMC, 50/50 (wt/wt), LP 50, Selectilyte®, BASF) was used as a reference system to compare the electrochemical performance.

### 2.2 Cell assembly and cycling procedure

The charge/discharge aging experiments in this work were performed using a CR 2032 coin cell setup with electrode areas of 1.13 cm^2^ for both anode and cathode. In addition to that, the cell chemistry consisted of MCMB/HC (MTI Corp.) and LiNi_0.6_Co_0.2_Mn_0.2_O_2_ (“NCM622”, Toda Kogyo Corp.) anode and cathode, respectively, as well as the electrolytes mentioned in Section 2.1. Both electrode types were manufactured with a pilot plant in-house and had an active mass of 10.98 mg and 14.25 mg for anode and cathode. Moreover, the cell preparation was performed in a water-free atmosphere by separating the positive and negative electrode using a separator consisting of one layer each of Celgard 2500 (Celgard LLC) and Freudenberg 2190 (Freudenberg SE) soaked with 100 μL electrolyte.

The constant current cycling investigations were performed using a Maccor battery test system (Maccor series 4000, Maccor Inc.) and varying charging rates (C-rates) of C/5 (32 mA g^−1^) and C/10 (16 mA g^−1^) (charges per hour) for the formation steps and C/1 (160 mA g^−1^) for the following cycles. Combining the formation and the subsequent cycles, procedures between 1 and 13 cycles were applied. Above that, storage tests for two weeks at room temperature were performed between the lower and upper cut-off voltage of 3.0 V and 4.3 V.

### 2.3 Sample preparation

After charge/discharge cycling, the coin cells were disassembled, the electrodes washed four times using 0.5 mL dimethyl carbonate (DMC, ≥99%, Sigma-Aldrich) followed by a drying procedure under reduced pressure at 60 °C. The separators were centrifuged at 8500 rpm using a Galaxy 5D (VWR LLC) to collect parts of the electrolyte for further analyses.

For the ICP-MS measurements of the aged electrodes, the samples were dissolved. This was done by microwave-assisted acid digestion using a Multiwave Pro (Anton Paar GmbH). Polytetrafluoroethylene (PTFE) liner were filled each with 3 mL nitric acid (65 vol%, Suprapur®, Merck KGaA) and hydrochloric acid (32 vol%, Suprapur®, Merck KGaA) followed by a 7 min power ramp to 1400 W, a hold period of 42 min and a cool down step until 55 °C was reached. Afterward, the samples were diluted using deionized water (2 μg L^−1^ TOC, 25 MΩ, MilliQ, Merck KGaA).

### 2.4 ICP-MS parameters

For all measurements, an Agilent 7900 ICP-MS (Agilent Technologies Inc.) was used. The main parameters were 1550 W for the RF power, 15.00 L min^−1^, 2.48 L min^−1^ and 2.00 L min^−1^ for the plasma, auxiliary and nebulizer gas flow, respectively. The sampling depth was set to 8 mm. Moreover, a MicroMist® nebulizer and a double-pass Scott-type quartz spray chamber were used.

### 2.5 GD-SF-MS measurements

An Element™ GD Plus (Thermo Fischer Scientific Inc.) sector field system was used in a pulsed DC operation mode with two different methods to obtain comparable sputter-rates for both sample types (anode and cathode). The method for the anodes was already reported in the literature.^47^ This method was adapted for the cathodes regarding the pulse conditions which was changed to 40 μs for the pulse duration and 1 kHz for the pulse frequency.

### 2.6 Determination of lithium fractions

In this work, eqn (1) is introduced and converted into eqn (2) in order to transform the measured ^6^Li-abundances into a separation of two different lithium fractions, the electrolytic and the cathodic fraction, depending on the calculated origin of the lithium.

1 *y*% = *x* × 95% + (1 − *x*) × 7.59%

2
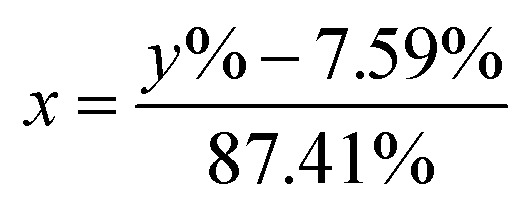


In these equations, *y*% represents the measured ^6^Li-abundance of the component, *x* stands for the electrolytic and (1 − *x*) for the cathodic lithium fractions. The percentages 95% and 7.59% in the equations are the enriched and natural abundance of ^6^Li, respectively.

## 3 Results and discussion

In the following the results of stored, formed and short-time aged cells are presented and discussed, whereas, no differentiation between metallic (plating) or ionic (degradation products) lithium on the electrode was made.

### 3.1 Benchmark measurements

**Fig. 1:**
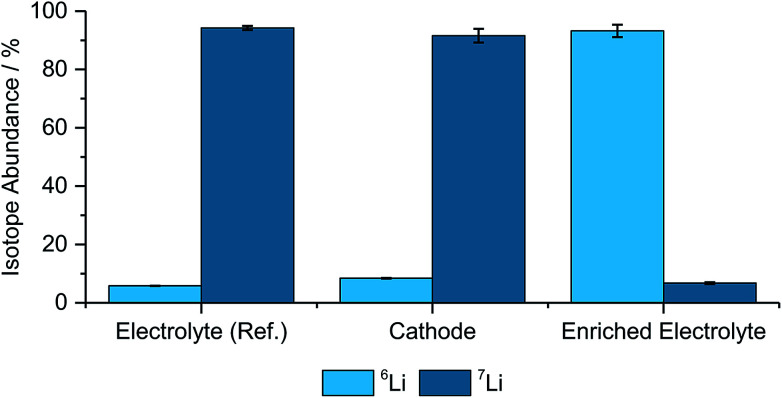
Determined isotope abundances for the different pristine materials including the two used electrolytes, a cathode, (*N* = 3).

Benchmark measurements were performed to determine the accuracy of determining the lithium-abundances in pristine components. Hence, NMC622, an enriched electrolyte, as well as the reference electrolyte were measured and compared with the natural abundance^48^ and the manufacturer's data from the enriched salt, which is seen in Fig. 1 and Table 1.

**Table 1:** Measured values for the isotopic abundances of ^6^Li and ^7^Li for the pristine cathode, the enriched (^6^LiPF_6_ as conducting salt) and reference electrolyte (^7^LiPF_6_ as conducting salt), (*N* = 3)

Component	^6^Li-abundance	^7^Li-abundance
Electrolyte (ref.)	5.79% ± 0.05%	94.21% ± 0.71%
Cathode	8.40% ± 0.17%	91.60% ± 2.33%
Enriched electrolyte	93.25% ± 2.12%	6.75% ± 0.33%

As depicted in Fig. 1 and Table 1 the abundances are matching well with the theoretical values, however, the deviations, especially for the cathode and the enriched electrolyte part, are significant. However, due to the differences between the natural and enriched abundances, small changes in the isotope ratios can still be resolved and qualitative analyses can be performed with these techniques.

### 3.2 ICP-MS measurements of aged LIB cells

In Table 2, the lithium ratios for the ^6^Li isotope for anode, cathode, and electrolyte are shown after a two-week storage test, one formation step at C/5 (32 mA g^−1^) and 13 cycles at C/5 and C/10 (16 mA g^−1^) for the formation and a C-rate of C/1 (160 mA g^−1^) for the subsequent cycles.

**Table 2:** Total amounts of ^6/7^Li in the pristine materials according to their respective masses and concentrations from Sections 2.1 and 2.2 as well as the ^6^Li-abundances of the different aged components of discharged cells after several cycling procedures and storage tests, (*N* = 3)

Component	Total amount of ^6^Li/^7^Li/mg	Storage (2 weeks)/%	1 Form. step/%	Formation C/5 + 10 cycles/%	Formation C/10 + 10 cycles/%
Anode	<LOD	<LOD	43.67 ± 1.04	45.02 ± 1.91	36.13 ± 1.60
Cathode	0.079/0.866	6.77 ± 0.30	31.04 ± 0.85	37.31 ± 2.01	35.61 ± 0.35
Electrolyte	0.567/0.041	91.09 ± 0.55	49.56 ± 0.64	43.42 ± 2.30	50.20 ± 1.57

The results show that the storage tests had only a minor influence on the measured isotope ratios. There was no lithium detectable on the digested anodes which could be explained due to the applied washing and drying processes removing most of the electrolyte in the wetted pores, as well as, no potential was applied to the tested cells that could cause a decomposition of the electrolyte followed by the formation of an SEI and CEI. Additionally, small changes of the ^6^Li-abundances were detected for the electrolyte and the cathode in comparison to the benchmark measurements.

However, this trend changes after the first charge/discharge cycle, where the abundances of the bulk materials showed a significant alteration compared to the benchmark. In detail, the ^6^Li abundance of the cathode rose to 31.04% from 8.40% and decreased from 93.25% to 49.56% for the electrolyte. Moreover, the first measurable ^6^Li-abundance on the anode started at 43.67%. Considering all these data, there is a strong tendency towards the assumption that the electrolyte plays an active role in the intercalation and de-intercalation processes in lithium ion batteries because these measurements showed that lithium from the electrolyte gets incorporated into the structure of the cathode and *vice versa* yet after the first formation step.

Furthermore, after ten cycles using a rate of C/5, the ^6^Li-abundances did not show significant changes compared to cells cycled for one formation step. Especially for the anode, the differences were only 1.35%. This implied that the formed SEI during the first cycle is effective, hence, not much additional lithium from cathode or electrolyte is lost during subsequent cycling. The ^6^Li-abundance on the cathode rose to 37.31% and decreased to 43.42% in the electrolyte, which could be explained by the increased probability of ^6^Li-ions intercalation into the lattice structure due to prolonged cycling (intercalation and de-intercalation).

By using a lower C-rate during the formation, the ^6^Li-abundance lowers especially on the anode to 36.13% and increased to 50.20% for the electrolyte, explained by longer diffusion times – 10 hours compared to 5 hours – of lithium from the cathode towards the anode, hence, leading to the formation of the anode SEI that contains higher lithium fractions.

### 3.3 Depth profile analysis using GD-SF-MS

**Fig. 2:**
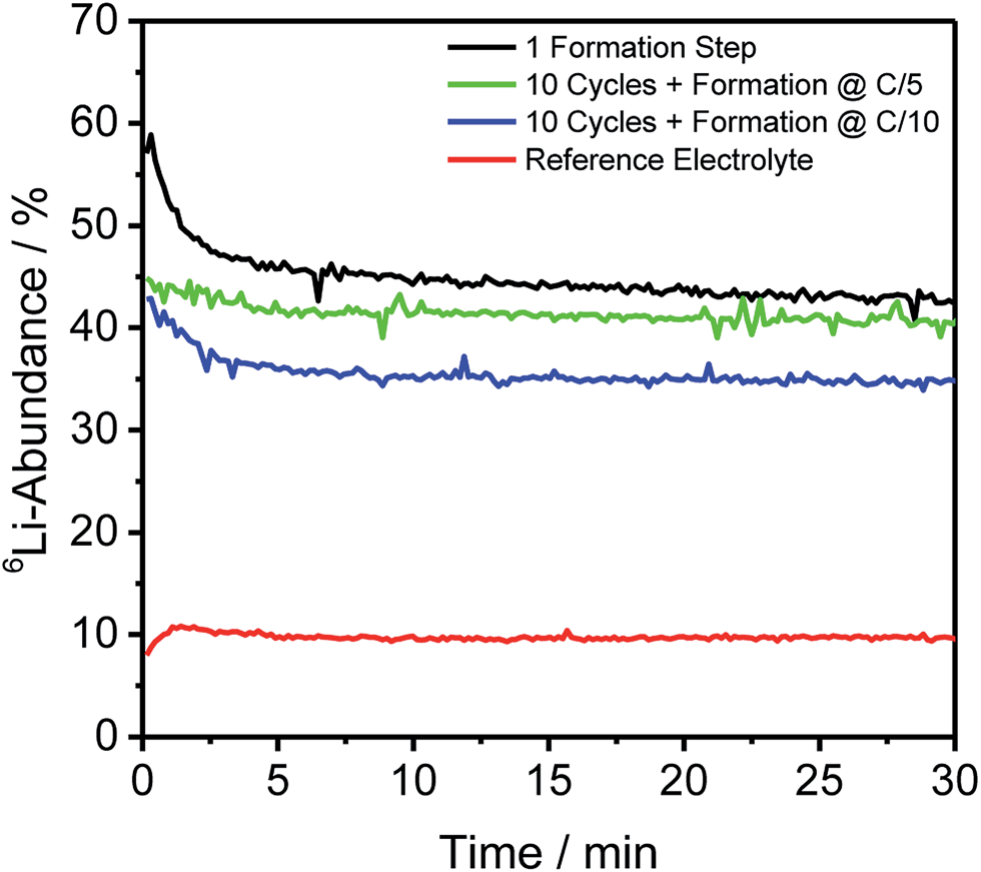
Depth profiles of four different discharged anodes aged under different cycling procedures including one formation step with the enriched and reference electrolyte and ten cycles with different C-rates during formation. The storage test is missing due to no significant amount of lithium being detectable.

For depth-resolved insights into the distribution and composition of lithium on the anode and cathode, respectively, GD-SF-MS measurements were performed for cells aged under the same conditions as described in Section 2.2. The ^6^Li-abundances for all anodes, as well as the reference cell, are shown in Fig. 2, except the storage test because no measurable change in the lithium abundance was detected.

Fig. 2 shows similar trends as already discussed for Table 2 regarding the bulk isotope abundances. However, this technique has the additional benefit of revealing the distribution throughout the profile of the electrode and in this case an agglomeration in the first minutes of the measurement of ^6^Li was detected to a certain degree for all cells using the enriched electrolyte.

In detail, the overall abundance of ^6^Li is the highest after the first formation step and atop on that, the ^6^Li-abundance started at 57.45% and has a plateau-value of 43.88%. This is in good correlation with the findings from ICP-MS bulk measurements and the difference of the ratio is less than 0.2% compared to the bulk material. Using the same C-rate for the formation but adding ten more charge/discharge cycles leads to an average ^6^Li-abundance of 41.11% during the plateau from 6 minutes onward. Comparing this decrease of the abundances with the increase in Table 2, shows a difference between the two measuring methods, but may be explained to the fact that different cells had to be used for the measurements and the deviations could be caused due to the cycling of the cells.

On top of that, the differences for the C-rate C/5 and C/10 are in a comparable extent, with the cell formed with C/10 having a lower overall ^6^Li-abundance of 35.03% at the plateau, leading to comparable findings as described in Section 3.2 that the slower C-rate during the formation promoted the incorporation of ^7^Li (cathodic) into the SEI of the discharged anodes.

**Fig. 3:**
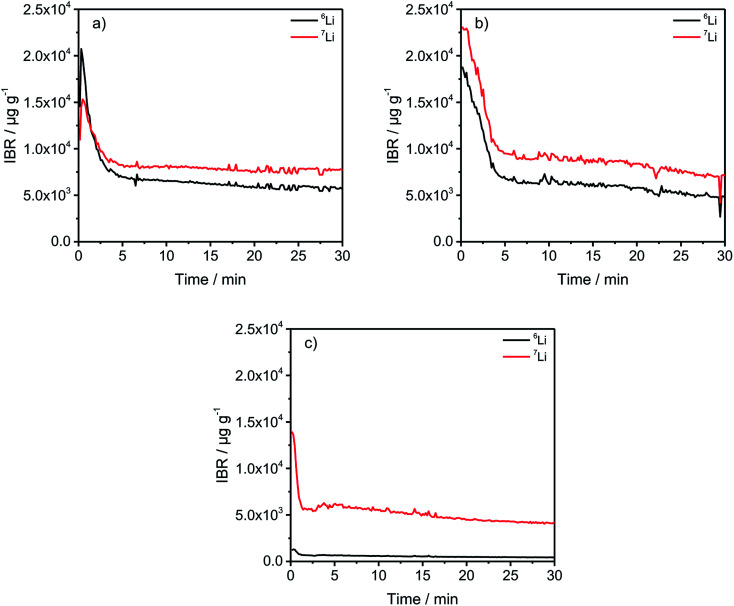
IBRs for the ^6^Li and ^7^Li signals of differently aged, discharged anodes. (a) one formation step with C/5; (b) three formation steps with C/5 plus ten additional cycles with C/1; (c) one formation step with C/5 and the reference electrolyte cycled using the same procedure as the enriched cells for one formation step.

In Fig. 2, a general trend is apparent in the first 5 minutes of each measurement in the form of an agglomeration of lithium on the surface of the anodes which may be due to lithium from the electrolyte that deposits directly after applying a voltage because of the direct contact with the negative electrode. While the data in Fig. 2 presented only qualitative considerations, it is also of utmost importance to compare the lithium signals semi-quantitatively. For this, the concentrations of the ^6^Li and ^7^Li isotopes were determined using the ion beam ratios (IBRs) with C, Li, and Cu as matrix elements and compared in Fig. 3 for three different cells (a) cycled for one formation step with a C-rate of C/5, (b) cycled for three formation steps at C/5 and ten subsequent cycles at C/1, and (c) cycled with the reference electrolyte for one formation step with a C-rate of C/5.

Fig. 3(c) shows a low bulk concentration of 0.53 mg g^−1^ for the total amount of lithium. The measurements were stopped before a complete depth profile through the active material was achieved. The reasoning is the constitution of the sample itself because the porous nature of the anodes leads to the detachment of parts of the carbonaceous material, revealing spots of the copper current collector which changes the sputter-rates and measured intensities and leads consequently to wrong results. Therefore, the measurements were stopped after 30 minutes as the intensities were almost constant.

The comparison of the two cells in Fig. 3(a) and (b) reveal on the one hand that the total lithium content in the bulk material is 1.39 mg g^−1^ for (a) and 1.41 mg g^−1^ for (b) if the averages of the combined contents of the ^6^Li and ^7^Li isotopes between 6 and 30 minutes are added together. This means that almost the same amount of lithium is lost after one formation step, as well as after the longer cycling procedure. However, during the first 5 minutes of sputtering-equals a depth of 2–3 μm – the ^6^Li signals are almost identical regarding the peak-, as well as plateau-values. But, the amount of ^7^Li on the surface of the anode, increased dramatically during the longer cycling procedure. While the highest measured concentration after one formation step is 1.53 wt%, it increased to 2.31 wt% after ten cycles, which equals an increase of 50.98% of the total lithium amount. Subsequently, this led to the assumption that lithium is accumulated predominantly on the surface of the anodes during prolonged charge/discharge cycling.

**Fig. 4:**
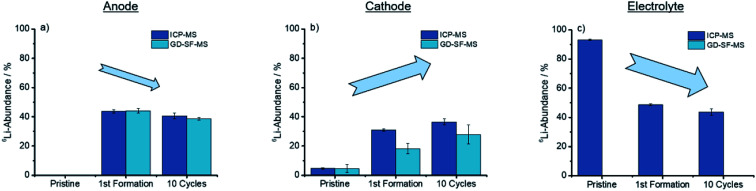
Trends showed after cycling for the discharged anode, cathode, and electrolyte for the ICP-MS, as well as the GD-SF-MS measurements.

As concluded in Fig. 4, both methods show the same trends for anode and cathode, while the deviation for the cathode is significantly higher.

From Fig. 4(a) a trend is apparent that the ^6^Li-abundance is slightly declining after the first formation step. The cathodes in Fig. 4(b) shows an opposite trend with an increase in the abundance, but deviations between the two analytical methods are over 10% of the lithium abundance for the first formation step, while the abundances for the pristine electrodes have a good fit towards each other.

### 3.4 Determining the lithium migration

**Fig. 5:**
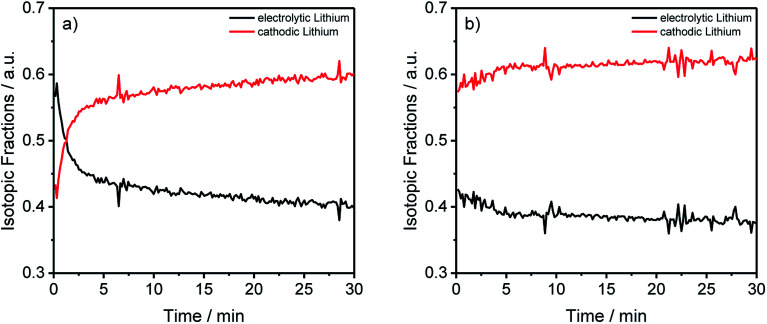
Lithium abundances from Fig. 2(a) and (b) transformed into the electrolytic and cathodic fractions for the different components and aging conditions.

In the following section, the lithium migration through the whole cell by converting the previous results with eqn (1) and (2) is presented. These results are depicted in Table 3 and Fig. 5 showing the lithium fractions from the two different lithium sources, in the following referred to as “electrolytic” and “cathodic” lithium.

**Table 3:** ^6^Li-abundances from Table 2 transformed into the electrolytic and cathodic fractions for the different components and aging conditions

	Anode		Cathode		Electrolyte	
	Electrolytic	Cathodic	Electrolytic	Cathodic	Electrolytic	Cathodic
Storage	<LOD	<LOD	0.00	1.00	0.96	0.04
1 form	0.41	0.59	0.27	0.73	0.48	0.52
10 Cy. C/5	0.43	0.57	0.34	0.66	0.41	0.59
10 Cy. C/10	0.33	0.67	0.32	0.68	0.49	0.51

In Table 3 two tendencies can be observed. First, all anodes show the trend that more than half of the deposited lithium originated from the cathode and the cells cycled at C/5 during the formation have almost the same electrolytic lithium fractions. A significantly lower fraction was achieved using C/10 during formation. Thus, the origin of the decomposed lithium is dependent on the charging rate, precisely, slower rates favor the deposition of cathodic lithium on the anode. Moreover, roughly two-thirds of the total lithium originated from the cathode for the cells cycled with C/10 during formation, assuring the presumption that a slower C-rate leads to a higher cathodic lithium fraction. The second trend was noticed for the cathode. In detail, a correlation between the electrolytic lithium fraction and the cycle number is shown in the data, leading to a higher fraction of Li after longer cycling procedures independent of the applied C-rates. For the electrolyte, no clear trend could be detected so far, which could be explained due to the surplus of electrolyte in coin cells compared to other cell setups such as cylindrical 18 650 cells.

By converting the concentration-dependent depth profiles in the same way as it was done for Table 3, the differences between the formation process in Fig. 5(a) and the longer cycling procedure in Fig. 5(b) gets even more apparent. While Fig. 5(b) shows an almost constant distribution of the electrolytic and cathodic lithium through the whole sputtered profile, a strong accumulation of electrolytic lithium on the surface of the measured anode in Fig. 5(a) was detected. Furthermore, this agrees with the previously discussed results from Fig. 3 where an agglomeration of ^7^Li on prolonged cycled anodes was detected.

The transformed results from the ICP-MS and GD-SF-MS measurements elucidate the importance of the applied isotope analysis with regard to migration and decomposition processes in LIBs, revealing that the electrolytic lithium plays a most active part in the migration. In detail, a fast mixing of cathodic and electrolytic lithium is happening during the first charge/discharge cycle. Moreover, it is also shown that a higher amount of lithium from the electrolyte is decomposed on the surface of the negative electrode, which could not be determined without the use of isotopic-labeled components.

## 4 Conclusion

The combination of different elemental analysis methods with an isotope analysis was presented and proved to be strong techniques for a fundamental implementation of plasma-based mass-spectrometric techniques and qualitative depth-resolved isotope analysis to determine the migration of lithium through the lithium ion battery cells during cycling. It was possible to determine the changes of the ^6^Li-abundances during cycling and to qualitatively compare the findings of the ICP-MS measurements with depth profile analyses using GD-SF-MS system. The measurement of two-week storage tests lead to only minor changes of the ^6^Li-abundances, but due to the small changes of the abundances and the general deviation of the measuring method, these changes are still in the margin of error of the method and could be repeated with longer storage times or with analytical techniques such as a multi-collector-ICP-MS (MC-ICP-MS) to improve the accuracy of the results.^49^ Both methods show the same trends for the measured abundances, while significantly higher deviations were observed for the cathodes measured using the GD system, leading to the exclusion of depth profiles in this work. Generally, the anodes show a slight decline of the ^6^Li-abundance from the first cycle onwards. For the cathodes, the opposite trend is shown with a steady increase of the abundances due to ^6^Li-ions that could be inserted into the layered structure during each discharge step. The measurements of the electrolyte revealed a strong decline of the enriched lithium abundance after the first cycle of 41.53% followed by a decline of 6.14% between this and cycle 13.

Since the mixing of the two different isotopic species happened to the biggest extent already after the first charge/discharge cycle and only slight changes could be observed after 13 cycles, further cycling experiments with longer cycling procedures were neglected in this work. Still, an influence of the different charge/discharge conditions could be determined with more cathodic lithium being incorporated into the SEI of the anode when a slower charging rate was applied during the formation which increased the time period in which the isotopic species could be mixed.

Moreover, by determining the fractions between lithium from the electrolyte and the cathode, so-called electrolytic and cathodic lithium fractions, it could be shown that the trends and tendencies are different for the three measured cell components. For the anodes, the fractions are particularly influenced by the charging rate during the formation because they show only minor differences if the same charging rate is applied but change between 13.6% and 17.5% for the cathodic fraction by bisecting the C-rate. The fractions for the cathode are influenced by the number of charge/discharge cycles leading to a higher electrolytic lithium fraction after a higher number of cycles. For the electrolyte, no clear trend is apparent which could be explained due to the surplus of electrolyte in coin cells in comparison to other cell setups. Above that, the GD depth profiles reveal that a strong accumulation of electrolytic lithium is detected on the surface of the anode after the first formation step. With a longer cycling procedure, this trend fades away and leads to almost constant lithium fractions throughout the electrode. This may be due to lithium from the electrolyte that decomposes directly after applying a voltage because of the direct contact with the negative electrode. All these findings considered, the conversion method, described in Section 2.6 to transfer the ^6^Li-abundances into the respective lithium fractions, is a strong technique to gather insights into the migration and distribution of lithium during cycling of LIB cells. As a follow-up to this work, these measurements should be extended using a SIMS system to gather additional information about the isotopic distribution on a particular scale.

## Conflicts of interest

The authors declare no conflict of interest.

## Supplementary Material
